# Increased susceptibility and volume reduction in deep brain nuclei in primary orthostatic tremor

**DOI:** 10.1136/bmjno-2025-001513

**Published:** 2026-03-02

**Authors:** Karolina af Edholm, Russell Ouellette, Mathias Sundgren, Erik Fransén, Anders Svenningsson, Tobias Granberg, Henrik Sjöström

**Affiliations:** 1 Department of Clinical Sciences Karolinska Institutet Danderyd Hospital, Karolinska Institutet, Stockholm, Sweden; 2 Neuroenheten Sabbatsberg, Stockholm, Sweden; 3 Department of Neuroradiology, Karolinska Institute, Stockholm, Sweden; 4 Department of Clinical Neuroscience, Karolinska Institutet, Stockholm, Sweden; 5 Department of Neurology, Karolinska University Hospital, Stockholm, Sweden; 6 School of Electrical Engineering and Computer Science, KTH Royal Institute of Technology, Stockholm, Sweden; 7 Digital Futures, KTH Royal Institute of Technology, Stockholm, Sweden; 8 Department of Clinical Science, Intervention and Technology, Karolinska Institutet, Stockholm, Sweden; 9 Department of Neurology, Danderyd Hospital, Stockholm, Sweden; 10 Centre for Neurology, Academic Specialist Centre, Stockholm, Sweden

**Keywords:** TREMOR, MOVEMENT DISORDERS, MRI

## Abstract

**Background:**

Primary orthostatic tremor (POT) is a rare progressive neurological disorder with a high-frequency tremor while standing. The pathophysiology remains uncertain, and imaging studies have been inconclusive.

**Objectives:**

The primary objective was to assess whether neurodegenerative changes are detectable in POT by quantitative susceptibility mapping (QSM) on brain MRI. A second objective was to explore volumetric changes in subcortical grey matter and cerebellum.

**Methods:**

In this cross-sectional study, 20 participants with POT and 14 age- and sex-matched healthy controls (HCs) were examined using a 3 T Siemens Prisma scanner with a T2*-weighted multiecho gradient-echo sequence for QSM. Regions of interest (ROIs) were automatically and manually segmented. Selected ROIs were the red nucleus, globus pallidus (GP), thalamus, caudatus, putamen, substantia nigra, dentate nucleus and inferior and superior colliculus.

**Results:**

There was a significant increase in susceptibility in the red nucleus (mean susceptibility 138.1 parts per billion (ppb) in POT and 113.3 ppb in HC, p=0.015) and GP (mean susceptibility 101.2 ppb in POT and 73.3 ppb in HC, p=0.0015) and a significantly lower volume of the GP in the POT group compared with HC (mean volume 3838 mm^3^ in POT and 4062 mm^3^ in HC, p=0.012).

**Conclusion:**

Increased susceptibility and decrease of volume indicate the possibility of a neurodegenerative process affecting the red nucleus and GP in POT. The red nucleus and GP are involved in motor control, and a focal dysfunction in these networks may be a part of the cause of orthostatic tremor.

**WHAT IS ALREADY KNOWN ON THIS TOPIC**

**WHAT THIS STUDY ADDS**

- This study shows an increased susceptibility in the red nucleus and globus pallidus (GP), and a lower volume in GP, changes that imply a neurodegenerative process in these areas.

**HOW THIS STUDY MIGHT AFFECT RESEARCH, PRACTICE OR POLICY**

- The red nucleus and GP are involved in motor control and are plausible sources of tremor generation. Our results highlight the possible involvement and a focal dysfunction of these structures in the pathogenesis of orthostatic tremor.

## Introduction

 Primary orthostatic tremor (POT) is a rare neurological disorder characterised by a fast tremor of 13–18 Hz in leg and trunk muscles while standing, and a strong sensation of imbalance that diminishes when the patient sits down or starts walking.^1 2^ POT has been considered a neurodegenerative progressive condition where patients report a decreasing ability of being in a standing position and gradual development of walking difficulties, while the tremor frequency remains stable over time.^3^ The tremor is transferred to the arms when the patient leans onto something or assumes a quadruped position.^4^ A tremor in the arms resembling essential tremor and Parkinsonian features are also common.^15^,^7^ However, POT does not seem to be a dopaminergic disorder according to the findings of normal levels of dopamine transporter in striatum, even though a few percent of patients respond to dopaminergic treatment.^5 8^ The pathophysiology of POT is unknown but is likely of central supraspinal origin and has been hypothesised to involve the network for stance and balance control involving cerebellum, brainstem, basal ganglia and supplementary motor area in cortex.^9^,^11^

In a retrospective study by Hassan *et al*,^5^ the medical records of 184 patients with POT were reviewed, out of which 135 patients had available MRI scans. The findings were mainly normal or showed age-related atrophy (40%), non-specific small vessel disease (30%), cerebral infarctions/lacunae (10%) and a few had incidental findings of tumours and local lesions. No consistent lesion location was found that could indicate the origin of the POT pathology.^5^

A few smaller radiological studies of POT have been conducted, where subtle changes in grey matter volume, functional connectivity, regional cerebral blood flow, regional cerebral glucose metabolism, metabolic changes and white matter changes have been observed.^1012^,^18^ These changes have mainly been associated with the cerebellum, but also with varying involvement of the thalamus, basal ganglia, supplementary motor area and motor cortex. Some of these studies have been based on the same patient material, why individual abnormalities may be over-represented.^15^,^18^ A few case reports have related the occurrence of orthostatic tremor to local lesions in the midbrain and spinal cord.^19^,^21^

A possible method of detecting subtle neuropathology is by measuring iron accumulation. Iron is essential for nerve function but may also induce inflammation through free radical oxygen species, which is why dysregulation of iron homeostasis is associated with neurodegenerative changes.^22 23^ Iron accumulation can be detected on MRI using susceptibility-weighted imaging, but its paramagnetic effects can shift surrounding tissue signals. Quantitative susceptibility mapping (QSM) minimises this artefact by analysing susceptibility voxelwise, providing higher definition. QSM is primarily used in research to assess group-level changes. Postmortem studies confirm its validity for measuring iron in deep grey matter, although white matter analysis is less accurate due to the diamagnetic effects of myelin.^24 25^

This study aimed to investigate whether susceptibility changes could be found in POT compared with healthy controls (HCs) using QSM from MRI data. We also evaluated group differences in the volumes of subcortical and cerebellar grey matter.

## Methods

### Study participants

Participants with POT were recruited from the cohort of the Study of Orthostatic Tremor in Sweden (SOTIS). The study was approved by the Swedish Ethical Review Authority (ref. no. 2020-01490, 2022-01802-02 and 2015/1607-31). The study was conducted in accordance with good clinical practice guidelines and the principles of the Declaration of Helsinki. All participants provided written informed consent.

Inclusion criteria in the POT group were previously diagnosed with POT according to the consensus statement of the classification of tremors.^2^ A control group of sex- and age-matched healthy individuals was included as HCs. This control group consisted of an HC group from a Parkinson’s disease MRI study cohort (KIMOVE), with an additional female control recruited for this study. Exclusion criteria for both patients and controls were medical conditions and metallic implants inappropriate for MRI scans. At the time of the MRI scans, participants with POT were assessed again for Parkinsonism (rest tremor, bradykinesia or rigidity according to the definition in Unified Parkinson’s Disease Rating Scale (UPDRS), part III) since patients with POT often present Parkinsonian features. All patients were also assessed with the Montreal Cognitive Assessment (MoCA), Schwab and England Activities of Daily Living (ADL) scale, Orthostatic Tremor Impact Profile (OTIP) and Orthostatic Tremor Severity and Disability Scale (OT-10) to assess the severity of POT.^26^,^29^ All participants with POT were neurologically examined by KaE.

### Data acquisition

MRI data were acquired using a Siemens MAGNETOM Prisma^fit^ 3 T scanner (Siemens Medical Systems, Erlangen, Germany). T_2_*-weighted gradient-echo sequence volumes were acquired using the following parameters: 0.69×0.69×1.4 mm, TE1 6 ms, 5 echoes, ΔTE 5.4 ms and TR 39 ms. High-resolution 3-D T1-weighted magnetisation-prepared rapid gradient-echo images were acquired for volumetric analysis; 1.0×1.0×1.0 mm, TE 2.52 ms, inversion time 900 ms and TR 1900 ms.

### QSM processing

Brain masks were created from the T_2_*-weighted volumes by use of FSL-BET on the average magnitude of the first three echoes.^30^ Quantitative susceptibility maps were generated using the morphology-enabled dipole inversion (MEDI) toolbox/MEDI+0, including Laplacian phase unwrapping, background phase removal using projection onto dipole fields and phase-to-source inversion with MEDI+0.^31 32^

### Volumetric analysis

Automated segmentation of brain structures was performed using FreeSurfer (V. 7.4.1, https://surfer.nmr.mgh.harvard.edu). All segmentations were manually quality checked and found to be without need of manual interventions. Volume statistics were extracted from the following regions of interest (ROIs); the thalamus, caudate nucleus, putamen, globus pallidus (GP), brainstem, cerebellar cortex and cerebellar white matter.

Normalisation was then performed using a method described by Voevodskaya *et al*,^33^ where the adjusted volumes were given as follows:


$$Volume_{adjusted}=Volume_{raw}-\beta^{*}(eTIV-eTIV_{mean})$$

where β is the slope of the regression line between the estimated total intracranial volume (eTIV, as given by the FreeSurfer segmentation) and the respective brain volume. For this normalisation, we used β-values and eTIV_mean_ derived from the control group and applied them to both groups.

### Segmentation of ROIs

The susceptibility maps were registered to the T1-volumes using EasyReg and SynthMorph (Learning Contrast-Invariant Registration Without Acquired Images).^34 35^ Susceptibility values from the caudate nucleus, GP, putamen and thalamus were extracted using the FreeSurfer segmentations. Manual segmentations were also performed (by HS) to delineate the red nucleus, substantia nigra pars compacta, dentate nucleus, superior colliculus and inferior colliculus on the QSM volumes using ITK-SNAP (V.4.0.0).^36^ Here, the two centremost axial slices of the respective structures were segmented.

### Data and statistical analysis

Statistics were calculated using SPSS (V.26, IBM Corp., Armonk, NY, USA). All ROIs were analysed as both sides (left+right) of each structure combined. Normality was assessed using the Shapiro–Wilk test. Possible differences between the groups with regards to age and sex were examined using Mann–Whitney U-test and χ^2^ test, respectively. Group differences in susceptibility and volume were tested using ANCOVA, with age and sex as covariates. Intrarater reliability for the susceptibility results in the manually segmented ROI was assessed in N=16 individuals, consisting of 8 randomly selected individuals from each of the two groups. This reliability analysis was performed using intraclass correlation coefficients (ICC) (two-way mixed model, absolute agreement and single measures), with 95% CIs. A p value<0.05 was considered significant.

## Results

### Clinical characteristics

A total of 20 patients with POT from the SOTIS cohort were included in the study, all presenting a rhythmic tremor activity of 13–18 Hz in standing position and no other neurological deficits. A control group of 14 healthy age- and sex-matched individuals was also included. Demographic data of all study participants and clinical features in the POT group are presented in table 1. There were no significant differences in age or sex distribution between the groups. Six of the participants in the POT group had mild Parkinsonian signs (a maximum of one point in each item in the UPDRS part III of uni- or bilateral rigidity or bradykinesia) but none had rest tremor or met the diagnostic criteria for Parkinson’s disease.^37^ All brain MRIs were neuroradiologically assessed. Besides age-related findings, this assessment also revealed extensive white matter changes suggestive of microangiopathy in one patient and several minor cerebellar infarcts in another. The mean disease duration was 14.4 years, 35% were regularly using walking aids and 80% were on tremor medication. 60% of the patients presented with arm tremor (postural or action tremor).

**Table 1:** Demography of study participants

Participants (n)	POT (20)	HC (14)	P value
Age (years) (median (IQR))	70 (16)	67 (15)	0.16
Sex (Female:Male)	15:5	7:7	0.13
Tremor frequency (Hz) (mean (SD))	15.3 (1.3)		
Disease duration (years) (mean (SD))	14.4 (9.5)		
Walking aid (n (%))	7 (35)		
Mild postural/action arm tremor (n (%))	12 (60)		
Mild Parkinsonian signs (n (%))	6 (30)		
OTIP (mean (SD))	50.6 (33.9)		
OT-10 (mean (SD))	23.6 (10.6)		
Schwab and England (mean (SD))	79.5 (13.6)		
MoCA (mean (SD))	25.9 (2.5)		

Group differences in age and sex were calculated with Mann–Whitney U-test and χ2 test, respectively. Results are presented as mean and SD.

HC, healthy control; MoCA, Montreal Cognitive Assessment; OT-10, orthostatic tremor severity and disability scale; OTIP, orthostatic tremor impact profile; POT, primary orthostatic tremor.

Patients had scores of moderate severity on the OTIP scale (50.6 of 188 points) and OT-10 scale (23.6 of 50 points), with high interindividual variability. Results from the Schwab and England ADL scale showed that participants were mainly independent in daily activities with a mean score of 79.5, where a score of 70 and lower is rated as dependent on assistance. Results from MoCA showed a mean score of 25.9, individual scores ranging from 21 to 29 (not adjusted for education or age) and one patient was later diagnosed with dementia during the following years.

### Group differences in QSM measurements

**Figure 1:**
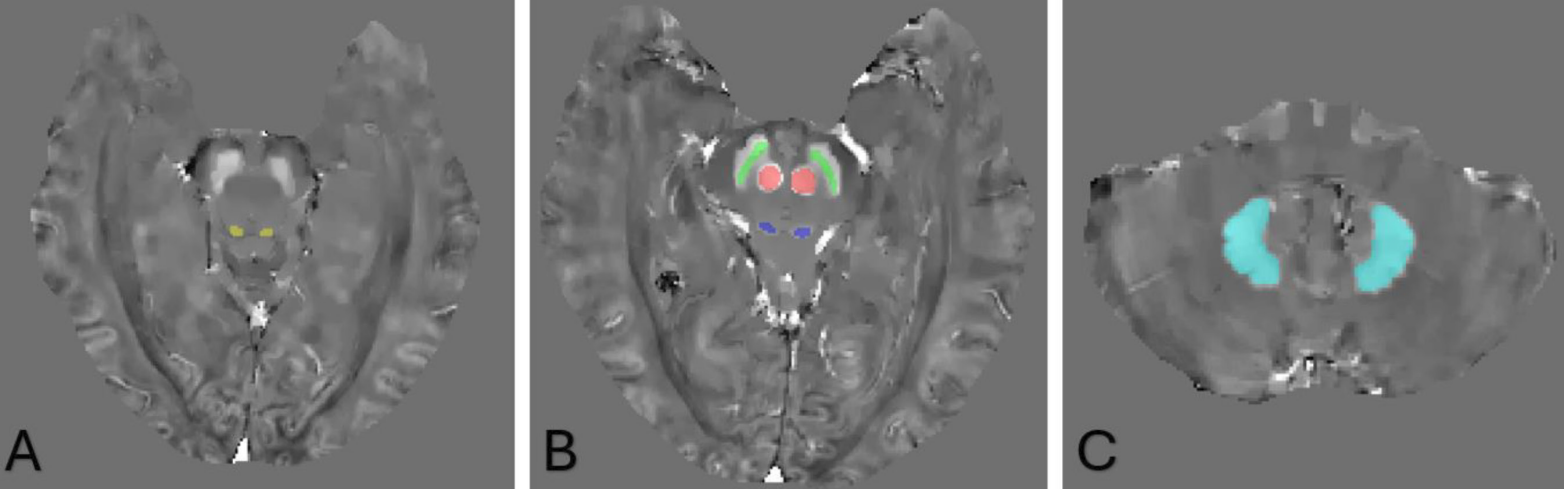
Visualisation of the manual segmentation of a male control in the mid 50s. (A) shows the inferior colliculus. (B) shows the substantia nigra pars compacta (green), the red nucleus (red) and the superior colliculus (blue). (C) shows the dentate nucleus.

**Figure 2:**
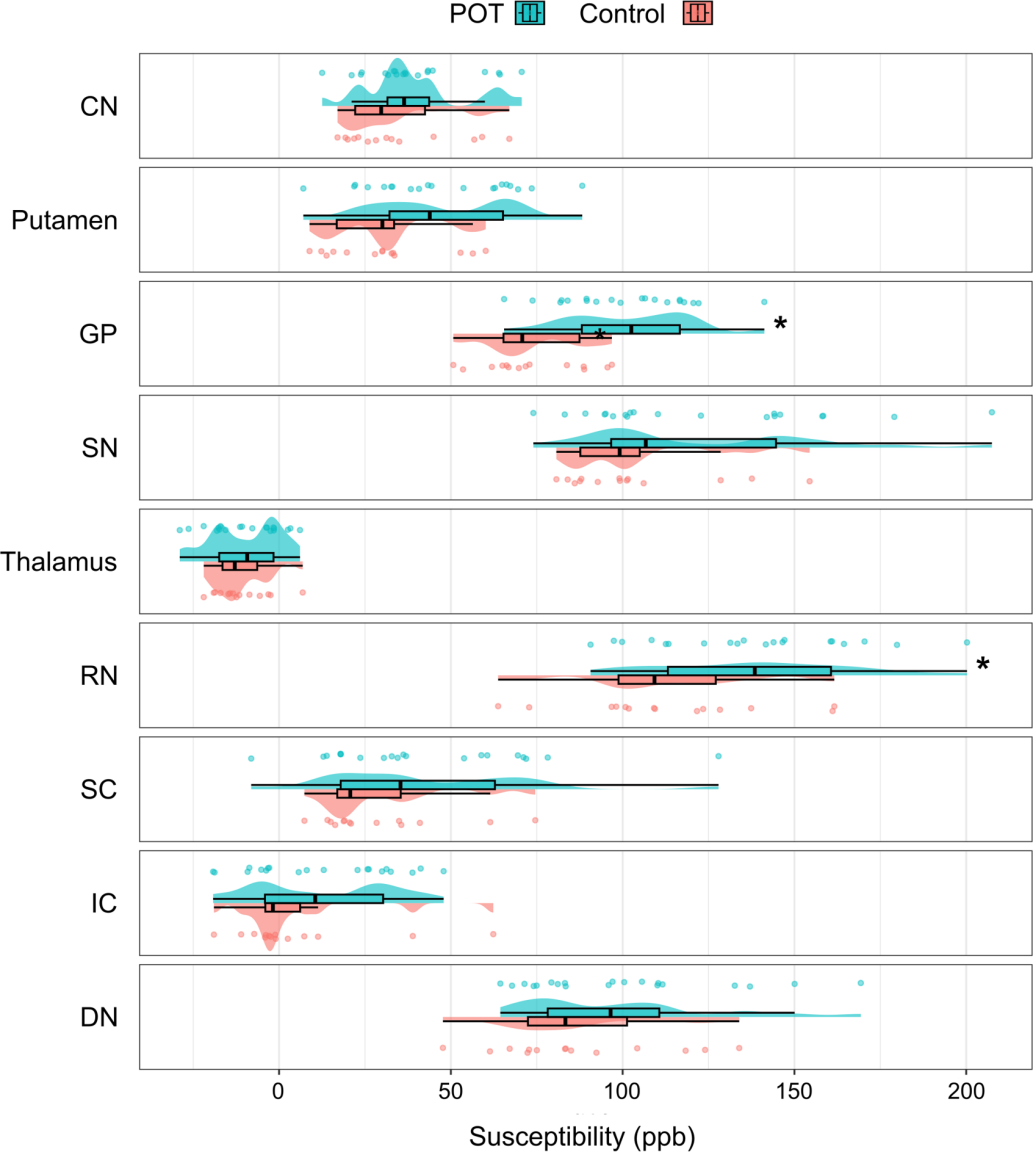
Group differences in susceptibility between patients with POT (green) and HCs (red). Asterisk (*) denotes significant group differences. CN, caudate nucleus; DN, dentate nucleus; GP, globus pallidus; IC, inferior colliculus; POT, primary orthostatic tremor; ppb, parts per billion; RN, red nucleus; SC, superior colliculus; SN, substantia nigra.

We found higher susceptibility in the red nucleus (p=0.015) and GP (p=0.001) in the POT group (with respective mean susceptibilities of 138.1 and 101.2 parts per billion (ppb)) compared with HCs (113.3 and 73.7 ppb, respectively), using ANCOVA with age and gender as covariates. Controlling for age and sex, the adjusted mean difference between groups was Δ=26.9 ppb (95% CI 5.6 to 48.2) in the red nucleus and Δ=20.8 ppb (95% CI 8.7 to 32.9) in the GP. The group effect size was partial eta squared (ηp²) = 0.187 in the red nucleus and ηp² = 0.298 in the GP. Visualisation of the manual segmentation of substantia nigra pars compacta, red nucleus, superior and inferior colliculus and the dentate nucleus is shown in figure 1. The susceptibility values in the respective groups are visualised in figure 2.

### Group differences in volumetric measurements

**Figure 3:**
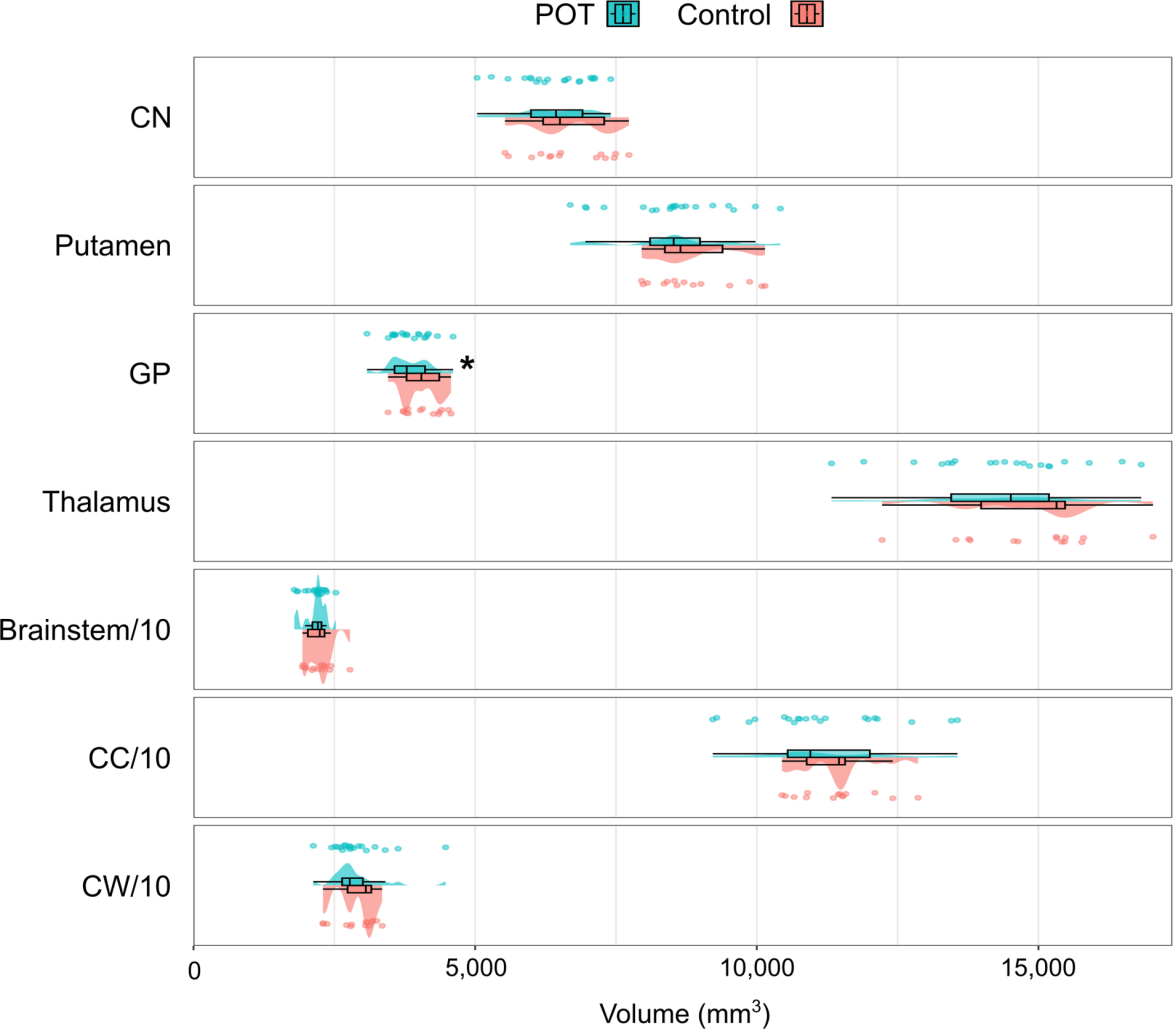
Group differences in volume between patients with POT (green) and HCs (red). Asterisk (*) denotes significant group differences. Note that the volume of the brainstem, cerebellar cortex and cerebellar white matter has been divided by 10 for presentation in this graph. CC, cerebellar cortex; CN, caudate nucleus; CW, cerebellar white matter; GP, globus pallidus; HCs, healthy controls; POT, primary orthostatic tremor.

We found a significantly lower volume in the GP in the POT group (mean volume 3838 mm^3^) compared with controls (4062 mm^3^) (p=0.012), assessed by ANCOVA with age and gender as covariates. Volume distributions are presented in figure 3.

### Sensitivity analysis with exclusion of individuals with vascular disease

With exclusion of the two individuals in the POT group with vascular disease (one with small cerebellar infarctions and one with pronounced white matter lesions), we still found higher susceptibility in the red nucleus (p=0.009) and GP (p=0.005), as well as lower volume of the GP (p=0.013) in the POT group compared with HCs, using ANCOVA with age and sex as covariates.

### Sensitivity analysis with visualisation stratified by sex

As an additional sensitivity analysis addressing the partial sex imbalance between groups, we visualised ROI values stratified by sex. For the three ROIs showing significant group differences in the primary analysis (red nucleus susceptibility, GP susceptibility and GP volume), box plots were generated separately for women and men in the POT and control groups (online supplemental figure 1). These sex-stratified distributions were assessed descriptively to evaluate whether the observed between-group differences were consistent within each sex. The distributions were broadly consistent with the primary findings, except for GP volume among women, where group medians and distributions appeared similar in both groups. Given the limited sample size within the sex strata, this analysis was intended as a qualitative robustness check rather than a formal interaction test.

### Intrarater reliability of manual segmentations

Repeated manual segmentations were performed by the same rater after a 6-month washout period on N=16 individuals (8 POT, 8 controls). For the manually segmented ROIs, intrarater reliability was high across all regions: red nucleus (ICC=0.998, 95% CI 0.996 to 0.999), substantia nigra (ICC=0.995, 95% CI 0.985 to 0.998), superior colliculus (ICC=0.991, 95% CI 0.976 to 0.997), inferior colliculus (ICC=0.993, 95% CI 0.965 to 0.998) and the dentate nucleus (ICC=0.985, 95% CI 0.959 to 0.995).

### Application of false discovery rate correction procedure to groupwise analysis

We also applied the false discovery rate correction procedure to closer examine the findings. With Benjamini–Hochberg adjusted p values, the finding of increased susceptibility in the GP remained significant (adjusted p=0.02). The findings of increased susceptibility in the red nucleus and decreased pallidal volume did not reach adjusted threshold (both adjusted p=0.08).

### Correlations to OT-10 scale

We assessed potential correlations between the OT-10 clinical symptom scale and the regions where significant group differences were found, using Pearson correlation test. No correlations were found between the OT-10 scale and the red nucleus or GP susceptibility values (p=0.644 and p=0.152, respectively). No correlation was found between the OT-10 scale and the GP volume (p=0.671).

## Discussion

In this cross-sectional study, we used QSM for quantifying levels of susceptibility in patients with POT compared with HCs. Relevant ROIs were chosen in the brainstem and basal ganglia for group comparison. Significant increases in susceptibility were observed in the red nucleus and GP in the POT group, which may reflect increased iron deposition or other susceptibility-altering tissue changes in these areas. Both the red nucleus and GP are nuclei involved in motor control and body adjustment. Given the exploratory nature of this study, we chose not to correct for multiple testings in our primary analysis. The findings should, therefore, be regarded as hypothesis generating and p values interpreted with caution. With an application of FDR correction, the finding of increased pallidal susceptibility remained significant, while the findings of increased red nucleus susceptibility and decreased pallidal volume did not reach adjusted significance threshold, although remaining relatively close to it.

The red nucleus is a primitive brainstem nucleus in the pontine tegmentum, consisting of a parvocellular part (RNp) and a magnocellular part (RNm). In humans, the red nucleus mainly consists of the RNp, which receives afferent information from cortex and the cerebellar dentate nuclei, and projects to the inferior olivary nuclei as a part of the cortico–rubro–olivo–cerebellar loop and the triangle of Guillain and Mollaret (red nucleus, ipsilateral inferior olive and contralateral dentate nucleus). The RNm has a major role in more primitive animals where projections to the rubrospinal tract are essential for locomotion, while in humans, this is largely replaced by the pyramidal tracts and the RNm is only rudimentary.^38^ The enhanced susceptibility found in this study may be consistent with a subtle neurodegenerative process in the RNp. This is further supported by the findings of Schoberl *et al*, where hypermetabolism was reported in pontine tegmentum in patients with POT.^13^ RNp projects inhibitory GABAergic stimulation to the inferior olivary nuclei, which has an internal neuronal pacemaker activity.^39^ Dysfunction in RNp may lead to alteration of the inhibitory stimulation of the inferior olivary nuclei and could result in a potential tremor generator.^39^

We also found increased susceptibility along with decreased volume of the GP in patients with POT. The GP plays an important part of the basal ganglia in planning and adjusting movements, where it receives input from striatum and projects to thalamus (the direct pathway) and the subthalamic nucleus (the indirect pathway). Efferent projections from the GP are mainly inhibitory, and dysfunction of the GP may result in tremor and dystonia. Although its name originates from the Latin word for pale, an effect of numerous transverse myelinated axons, GP still contains a high amount of iron and is a suitable structure for QSM analysis. The GP is divided into an external (GPe) and an internal part (GPi), where the GPi modulates the direct pathway projecting to thalamus, and GPe modulates the indirect pathway projecting to the subthalamic nucleus and then back to the substantia nigra and striatum. In our study, we did not see any difference in susceptibility between the GPi and GPe. Some of the previous explorative studies of POT have also reported findings in the GP. Benito-Leon *et al* found an age-correlated smaller volume of GP in patients with orthostatic tremor and Parkinsonian signs.^18^ Wills *et al* found an increase in blood flow in the lentiform nuclei (GP and putamen) in a positron emission tomography study of POT.^12^

We did not find any differences between POT and HC in cerebellar cortex, cerebellar white matter or the dentate nucleus even though varying alterations in volume, functional connectivity, blood flow and metabolism in cerebellum have been reported previously.^10 12 14 16^ These conflicting results between studies may be caused by the small number of participants, a recurrent problem in studies of rare diseases and the unclear aetiology of the POT symptomatology.

Our study has the limitation of a small patient population with a notable, though not significant, imbalanced sex distribution between the groups. However, results from sensitivity analysis did not suggest that sex had a major impact on susceptibility. The quantitative trait of QSM allows for comparison over time and between MRI scanners, which is why future larger studies could potentially be conducted. The methods we used did not allow us to segment the different cerebellar lobules, which may also have hidden possible relevant correlations in these regions. Iron accumulation may be increased by other pathologies, and stricter exclusion criteria could have been used to exclude participants with comorbidities associated with iron accumulation, for example, diabetes, cognitive impairment, vascular disease and Parkinsonian signs. Orthostatic tremor with additional neurological signs or neurological comorbidities, often referred to as orthostatic tremor plus, may also have different pathophysiologies. Separation between pure POT and orthostatic tremor plus in these kinds of studies may further improve our understanding. It could be speculated that the dysfunction of the GP, a structure also involved in associative and limbic circuitries, may be related to the reported mild cognitive impairment in POT.^40^ Also, as the study is cross sectional, QSM and volumetric differences cannot by themselves establish an ongoing neurodegenerative process. Finally, we cannot exclude the possibility of our findings being spurious observations from comparing several areas in a rather small cohort. Arguing against this is the known functions of the observed areas being involved in motor control.

## Conclusion

In this study, we found an increased susceptibility in the red nucleus and the GP, and a lower volume in GP, changes that are consistent with a possible neurodegenerative process in these areas. The red nucleus and the GP are involved in motor control and are plausible sources of tremor generation. These results support the plausibility of a focal dysfunction of these structures as a contributing cause of orthostatic tremor, but this needs to be confirmed in future studies.

## Supplementary material

10.1136/bmjno-2025-001513 online supplemental file 1

## Data Availability

Data are available on reasonable request.

## References

[1] Piboolnurak P, Yu QP, Pullman SL (2005). Clinical and neurophysiologic spectrum of orthostatic tremor: case series of 26 subjects. Mov Disord.

[2] Bhatia KP, Bain P, Bajaj N (2018). Consensus Statement on the classification of tremors. from the task force on tremor of the International Parkinson and Movement Disorder Society. Mov Disord.

[3] Ganos C, Maugest L, Apartis E (2016). The long-term outcome of orthostatic tremor. *J Neurol Neurosurg Psychiatry*.

[4] Boroojerdi B, Ferbert A, Foltys H (1999). Evidence for a non-orthostatic origin of orthostatic tremor. *J Neurol Neurosurg Psychiatry*.

[5] Hassan A, Ahlskog JE, Matsumoto JY (2016). Orthostatic tremor: Clinical, electrophysiologic, and treatment findings in 184 patients. Neurology (ECronicon).

[6] Mestre TA, Lang AE, Ferreira JJ (2012). Associated movement disorders in orthostatic tremor. *J Neurol Neurosurg Psychiatry*.

[7] Gerschlager W, Münchau A, Katzenschlager R (2004). Natural history and syndromic associations of orthostatic tremor: a review of 41 patients. Mov Disord.

[8] Trocello J-M, Zanotti-Fregonara P, Roze E (2008). Dopaminergic deficit is not the rule in orthostatic tremor. Mov Disord.

[9] Deuschl G, Raethjen J, Lindemann M (2001). The pathophysiology of tremor. Muscle Nerve.

[10] Gallea C, Popa T, García-Lorenzo D (2016). Orthostatic tremor: a cerebellar pathology?. Brain (Bacau).

[11] Benito-León J, Domingo-Santos Á (2016). Orthostatic Tremor: An Update on a Rare Entity. Tremor Other Hyperkinet Mov (N Y).

[12] Wills AJ, Thompson PD, Findley LJ (1996). A positron emission tomography study of primary orthostatic tremor. Neurology (ECronicon).

[13] Schöberl F, Feil K, Xiong G (2017). Pathological ponto-cerebello-thalamo-cortical activations in primary orthostatic tremor during lying and stance. Brain (Bacau).

[14] Phipps CJ, Whitney D, Shou J (2024). Measurement of Functional Brain Network Connectivity in People with Orthostatic Tremor. Brain Sci.

[15] Benito-León J, Romero JP, Louis ED (2019). Diffusion tensor imaging in orthostatic tremor: a tract-based spatial statistics study. Ann Clin Transl Neurol.

[16] Benito-León J, Louis ED, Mato-Abad V (2016). In vivo neurometabolic profiling in orthostatic tremor. Medicine (Baltimore).

[17] Benito-León J, Louis ED, Manzanedo E (2016). Resting state functional MRI reveals abnormal network connectivity in orthostatic tremor. Medicine (Baltimore).

[18] Benito-León J, Louis ED, Mato-Abad V (2019). A data mining approach for classification of orthostatic and essential tremor based on MRI-derived brain volume and cortical thickness. Ann Clin Transl Neurol.

[19] Vetrugno R, D’Angelo R, Alessandria M (2010). Orthostatic tremor in a left midbrain lesion. Mov Disord.

[20] Benito-León J, Rodríguez J, Ortí-Pareja M (1997). Symptomatic orthostatic tremor in pontine lesions. Neurology (ECronicon).

[21] Lee HM, Kwon D-Y, Park MH (2012). Symptomatic orthostatic tremor with progressive cognitive impairment in spinal cord lesions. Clin Neurol Neurosurg.

[22] Gao G, You L, Zhang J (2023). Brain Iron Metabolism, Redox Balance and Neurological Diseases. Antioxidants (Basel).

[23] Levi S, Ripamonti M, Moro AS (2024). Iron imbalance in neurodegeneration. Mol Psychiatry.

[24] Langkammer C, Schweser F, Krebs N (2012). Quantitative susceptibility mapping (QSM) as a means to measure brain iron? A post mortem validation study. Neuroimage.

[25] Sun H, Walsh AJ, Lebel RM (2015). Validation of quantitative susceptibility mapping with Perls’ iron staining for subcortical gray matter. Neuroimage.

[26] Nasreddine ZS, Phillips NA, Bédirian V (2005). The Montreal Cognitive Assessment, MoCA: a brief screening tool for mild cognitive impairment. J Am Geriatr Soc.

[27] Schwab J, England A Projecting technique for evaluating surgery in parkinson’s disease.

[28] Vijiaratnam N, Sirisena D, Paul E (2018). Measuring disease progression and disability in orthostatic tremor. Parkinsonism Relat Disord.

[29] Merola A, Torres-Russotto DR, Stebbins GT (2020). Development and Validation of the Orthostatic Tremor Severity and Disability Scale (OT-10). Mov Disord.

[30] Smith SM (2002). Fast robust automated brain extraction. Hum Brain Mapp.

[31] Liu T, Khalidov I, de Rochefort L (2011). A novel background field removal method for MRI using projection onto dipole fields (PDF). NMR Biomed.

[32] Liu Z, Spincemaille P, Yao Y (2018). MEDI+0: Morphology enabled dipole inversion with automatic uniform cerebrospinal fluid zero reference for quantitative susceptibility mapping. Magn Reson Med.

[33] Voevodskaya O, Simmons A, Nordenskjöld R (2014). The effects of intracranial volume adjustment approaches on multiple regional MRI volumes in healthy aging and Alzheimer’s disease. Front Aging Neurosci.

[34] Iglesias JE (2023). A ready-to-use machine learning tool for symmetric multi-modality registration of brain MRI. Sci Rep.

[35] Hoffmann M, Billot B, Greve DN (2022). SynthMorph: Learning Contrast-Invariant Registration Without Acquired Images. IEEE Trans Med Imaging.

[36] Yushkevich PA, Piven J, Hazlett HC (2006). User-guided 3D active contour segmentation of anatomical structures: significantly improved efficiency and reliability. Neuroimage.

[37] Postuma RB, Berg D, Stern M (2015). MDS clinical diagnostic criteria for Parkinson’s disease. Mov Disord.

[38] Stacho M, Häusler AN, Brandstetter A (2024). Phylogenetic reduction of the magnocellular red nucleus in primates and inter-subject variability in humans. Front Neuroanat.

[39] Lang EJ, Handforth A (2022). Is the inferior olive central to essential tremor? Yes. Int Rev Neurobiol.

[40] Benito-León J, Louis ED, Puertas-Martín V (2016). Cognitive and neuropsychiatric features of orthostatic tremor: A case-control comparison. J Neurol Sci.

