# Supplementary figures and images for: Increased susceptibility and volume reduction in deep brain nuclei in primary orthostatic tremor
Source: BMJ Neurol Open. 2026 Mar 2;8(1):e001513. doi: 10.1136/bmjno-2025-001513 (PMC12970075; doi:10.1136/bmjno-2025-001513)

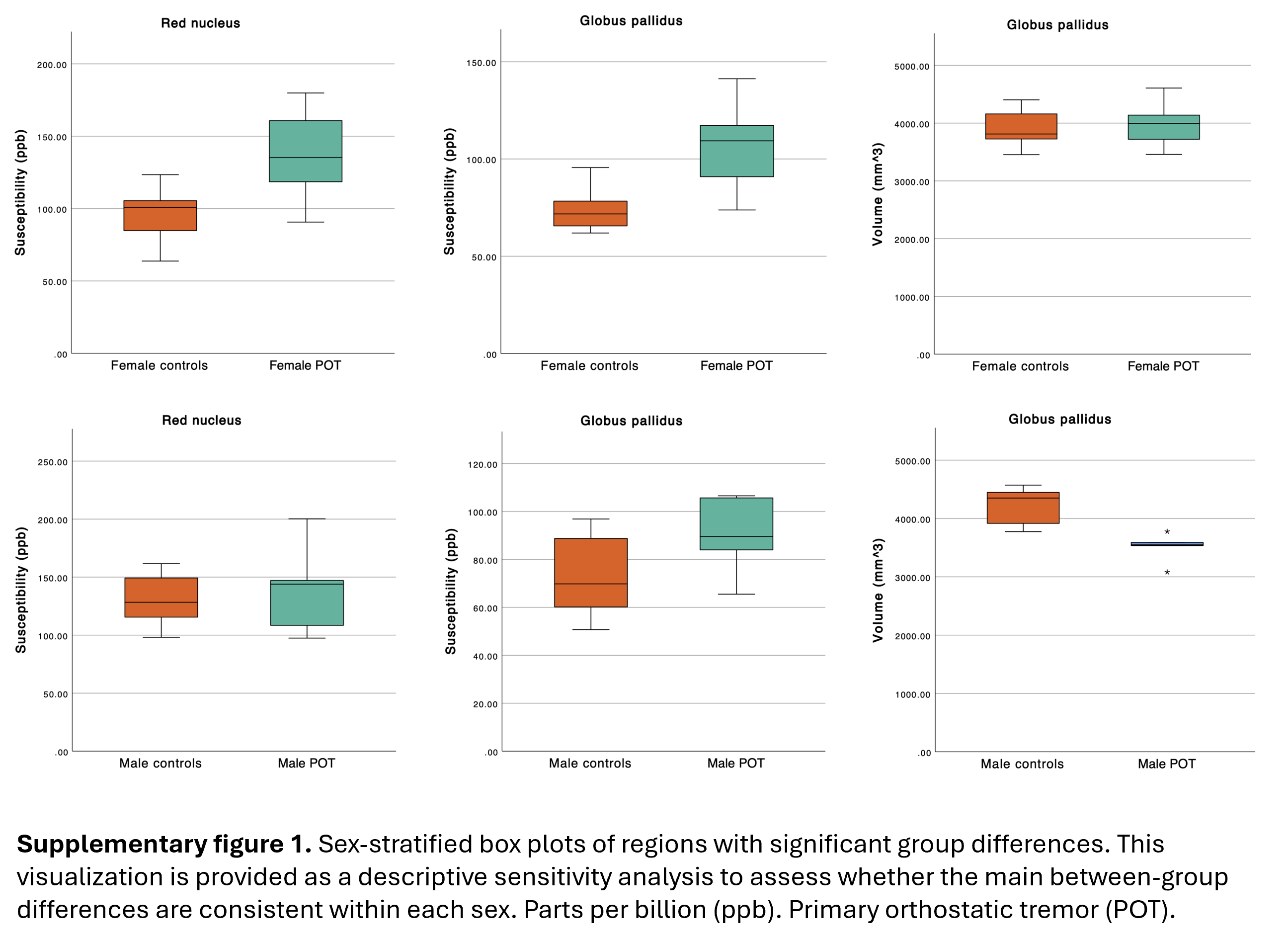

Supplement: online supplemental file 1 [file bmjno-8-1-s001.tif]
